# Social Cognition in Suicidal Behavior in Psychosis: A Systematic Review

**DOI:** 10.3390/bs15060759

**Published:** 2025-06-01

**Authors:** María Carcedo Herrero, Aina Sastre-Buades, Maria Luisa Barrigón

**Affiliations:** 1 Department of Psychology, Universidad a Distancia de Madrid (UDIMA), 28400 Collado-Villalba, Spain; mariacarcedoherrero@gmail.com (M.C.H.); aina.sastre@hsll.es (A.S.-B.); 2 Private Practice, 48008 Bilbao, Spain; 3 Department of Psychiatry, Son Llàtzer University Hospital, 07198 Palma, Spain; 4 Centro de Investigación Biomédica en Red de Salud Mental (CIBERSAM), 28029 Madrid, Spain; 5 Instituto de Psiquiatría y Salud Mental, Hospital General Universitario Gregorio Marañón, 28007 Madrid, Spain; 6 Gregorio Marañón Health Research Institute, 28009 Madrid, Spain; 7 School of Medicine, Universidad Complutense de Madrid, 28040 Madrid, Spain

**Keywords:** psychosis, psychotic disorder, suicidal behavior, suicide, social cognition

## Abstract

Suicide is a major concern worldwide, especially in psychotic disorders that have an increased risk for suicidal behavior (SB). There are many well-established risk factors for SB in psychosis. Still, others, such as the domains of social cognition (SC)—the theory of mind, social perception, emotional processing, and attributional style—are unclarified. We aim to review evidence on SC and SB in psychosis and clarify their relationship, examining the differences between SC domains and the potential mediating variables in this relationship and proposing that worse performance in regard to SC is related to a higher risk of suicide. We searched databases for papers on SC and SB published between 2009 and 2024, resulting in the 18 articles included in this systematic review. Individuals with psychotic disorders and SB showed better emotional processing for basic emotional recognition—although they performed poorly on more complex tasks—and exhibited greater empathy within the affective theory of mind. Cognitive biases associated with attributional style and increased distrust as part of social perception were also found. Our findings cannot establish a relationship with the cognitive theory of mind. So, further studies are needed to integrate all domains of SC in longitudinal studies and examine the mediating variables of these relationships.

## 1. Introduction

Suicide is one of the main public health problems worldwide, with over 700,000 people dying each year, making it a priority objective of the World Health Organization (WHO, 2021). In Spain, it is also treated as a major problem, since according to the latest data established by the National Institute of Statistics (INE), in 2023, it resulted in 4116 deaths by suicide, again placing it as the leading external cause of death in our country (INE, 2023). The WHO (2014) defines suicidal behavior as a continuum that extends from suicidal ideation to death by suicide, including suicidal planning and attempted suicide, and is the result of a complex interaction of biological, psychological, social, and environmental factors (Turecki et al., 2019). The inclusion of suicidal ideation in this continuum, as an aspect that involves cognitive changes or deficits (Keilp et al., 2013, 2014; Roca et al., 2019; Westheide et al., 2008), remains a complex issue. Although there are discrepancies among different authors, it has been concluded that suicidal ideation is associated with worse verbal learning in major depressive disorder (Lan et al., 2020) or impaired decision making (Westheide et al., 2008), and they could be considered a cognitive biomarker of suicide risk (Lan et al., 2020; Sastre-Buades et al., 2021). Among the diagnoses with higher rates of suicidal behavior, we find that severe mental disorders—among which psychotic disorders, major depressive disorder, and bipolar disorder stand out—have much higher rates than the general population, thereby increasing the risk by five to ten times (Fu et al., 2023). Despite the high risk of suicidal behavior in psychotic disorders, limited research has been conducted on this topic, partly because a diagnosis of psychotic disorder is often an exclusion criterion in studies on suicidal behavior (Villa et al., 2019).

Individuals with psychotic disorder have a life expectancy 15–20 years shorter than the general population, with suicide as the main cause, although they also have a higher mortality from natural causes due to comorbid factors. Taking into account mortality risk and associated risk factors, clinical practice is essential, as such risks must be addressed urgently from multiple levels (Correll et al., 2022). The lifetime risk of suicide in these individuals is 5% (Hor & Taylor, 2010); however, when suicide attempts are taken into account, this rate rises at the onset of the disorder to 45.9%, at 6 months to 38%, and over a lifetime, it would be around 26.8% (Lu et al., 2019). Over the years, research has identified different factors that are associated with an increased risk of suicidal tendencies in these individuals. These factors include depressive symptomatology, a higher number of both positive and negative psychotic symptoms, frequent hospitalizations, comorbid drug use, a psychiatric history, a family history of suicide attempts, being male and under 40 years of age, poor adherence to treatment, and previous suicide attempts (Cassidy et al., 2018; Yüksel et al., 2024). This elevated mortality is associated with risk factors for suicidal behavior. Many of these factors can be modified through clinical practice, particularly social cognition, which this paper focuses on (Correll et al., 2022).

Recent years have seen a growing interest in variables such as neurocognition or social cognition and their role in psychosis. Taking these factors into account allows us to propose new therapeutic approaches for individuals with psychotic disorders who are at risk of engaging in suicidal behavior. Social cognition refers to the mental operations through which we interpret, analyze, remember, and use information about the environment and relationships, and thanks to this, we deduce the emotions of others and put ourselves in their place to understand what they will think or how they will react to a given situation. There are discrepancies in the conceptualization of social cognition, and therefore the NIMH has sought to explain more operationally what social cognition encompasses by dividing it into different domains. The first domain is theory of mind, developed by Premack and Woodruff (1978), which refers to the ability to distinguish between one’s mental states and those of others, including the understanding of false beliefs, intentions of others, deceptions, or metaphors. Within theory of mind, it is possible to differentiate between a more affective theory of mind related to the affective state, emotion, or feeling of others, and a more cognitive part about the beliefs, thoughts, or intentions of others (Jarvis et al., 2024). The second domain is social perception, defined as the identification and interpretation of different social situations based on the rules and roles in the social context, that is, the perceptual processes involved in directing attention to social signals. Third, attributional style refers to how the causes of social events and the intentions of others are usually understood, and involves the study of biases. Fourth, emotional processing encompasses the skills or strategies related to the perception and use of emotional information, including the identification, creation, understanding, and management of emotions and nonverbal language (Green et al., 2008; Pinkham, 2014).

Several studies have demonstrated deficits in social cognition in individuals with psychotic disorders, particularly in the categories of social perception, emotional processing, and theory of mind (Fortuny et al., 2023; Healey et al., 2016; Kohler et al., 2010; Savla et al., 2013). These deficits appear to be associated with negative symptoms, subsequent clinical depression (Pelizza et al., 2020), and poorer perceived quality of life in domains such as physical and psychological health, social relationships, and environment (Martín et al., 2017). Moreover, evidence suggests that social cognition impairment occurs both in early psychotic episodes and in chronic patients, showing stability throughout the disorder (Bora & Pantelis, 2013; Healey et al., 2016). Other studies have examined theory of mind in isolation and concluded that its impairment could predict a suicide attempt (Canal-Rivero et al., 2017). In line with this, without taking into account clinical diagnosis, differences have been found between deficits in social cognition and different levels of suicidal risk. That is, greater impairment is detected in individuals with a history of suicide attempts in comparison with individuals with suicidal ideation, who show worse performance than the general population, suggesting that social cognition may serve as a clinical marker associated with greater or lesser suicidal risk (Comparelli et al., 2022). Regarding social perception and attributional style, few studies are available in this population (Healey et al., 2016).

However, other studies suggest that individuals with a history of suicide attempts may perform better on social cognition tasks, exhibiting a very low externalizing bias (attribution of positive events to external elements), similar to individuals with depressive disorder, and demonstrating better theory of mind performance (Sastre-Buades et al., 2023). Additionally, individuals with previous suicide attempts have been found to show better empathic performance (Ma et al., 2024). Similarly, another study by Dickhoff et al. (2021) reported that both individuals with previous suicide attempts and those with current suicidal ideation performed better on emotional face recognition and theory of mind tasks. This could suggest that the factor influencing suicidal behavior is not a deficit in social cognition, but rather an insufficient ability to regulate social stress as a result of increased sensitivity to social relationships, which is contrary to findings from other studies that have directly identified a deficit in this social perception (Fortuny et al., 2023; Healey et al., 2016; Kohler et al., 2010; Savla et al., 2013).

Current research on social cognition in this population has identified two contrasting perspectives. One perspective indicates a clear lack of social cognition that may directly or indirectly lead to a higher probability of suicide, while the other suggests that these individuals have better social cognition, which could trigger greater social stress leading to suicidal behavior.

The main objective of this study is to conduct a systematic review of the relationship between social cognition, and its distinct domains, and suicidal behavior in psychotic disorders. As secondary objectives, we examine whether differences exist between the specific domains that comprise social cognition and whether additional variables mediate this relationship. We hypothesize that social cognition performance is associated with increased suicidal behavior in individuals with psychotic disorders. Therefore, this review aims to clarify whether social cognition constitutes a risk factor for suicidal behavior, with the goal of optimizing and individualizing treatment approaches (Correll et al., 2022; WHO, 2021). In this review, different concepts along the suicidal behavior continuum are considered and clearly differentiated from one another (WHO, 2014).

## 2. Materials and Methods

The present review was conducted in accordance with the updated of Preferred Reporting Items for Systematic Reviews and Meta-Analyses (PRISMA) guidelines (Page et al., 2021).

### 2.1. Eligibility Criteria

The selection criteria for the articles were established based on the objective of this study; therefore, included studies were required to address social cognition in relation to suicidal behavior with in psychosis.

We adopted the WHO perspective, which defines suicidal behavior as a continuum ranging from suicidal ideation to death by suicide, taking into account and differentiating the various concepts along this continuum (WHO, 2014).

Social cognition refers to the mental operations by which individuals interpret, analyze, and use information from their environment. In this review, we included the following domains of social cognition: theory of mind, social perception, attributional style, and emotional processing (Green et al., 2008; Pinkham, 2014). Theory of mind, as developed by Premack and Woodruff (1978), is defined as the ability to distinguish between one’s own mental states and those of others. Social perception is defined as the identification and interpretation of different social situations based on social rules and roles. Attributional style refers to the way in which individuals understand the causes of social events and the intentions of others. Emotional processing encompasses the skills and strategies related to the perception, understanding, and use of emotional information.

In addition, only scientific publications after 2009 were included. Meta-analyses or systematic reviews that did not specifically assess suicidal behavior in the psychotic population, did not measure social cognition, or focused solely on neuroimaging tests or other biological markers were excluded.

### 2.2. Information Sources

An exhaustive search was conducted in several databases, including ProQuest (which searches eight databases, such as PsycInfo), Web of Science (which also examines multiple databases), PubMed (which includes Medline and other resources), and Scopus. In addition to these four databases, a search was performed in Google Scholar, an academic search engine oriented towards scientific content. The search was conducted between 8 April 2024 and 22 April 2024.

### 2.3. Search Strategy

The search was limited to scientific articles published within the last 15 years (2009–2024) in English or Spanish, using keywords such as social cognition, theory of mind, social perception, attributional style, suicidal behavior, and psychosis. Articles not available in English or Spanish were excluded. A second-level search was performed by reviewing the bibliographic references of studies identified in the primary search that were relevant to the aim of this review. The same inclusion and exclusion criteria were applied as in the primary search.

The final search strategy was as follows: (psychosis OR schizophrenia) AND (suicide attempt OR suicidal behavior OR suicide risk) AND (social cognition OR theory of mind OR social perception OR social cognition OR attributional bias OR emotional processing). MeSH terms were not used, as the search was not limited to PubMed, and the strategy was replicated across multiple databases.

**Figure 1:**
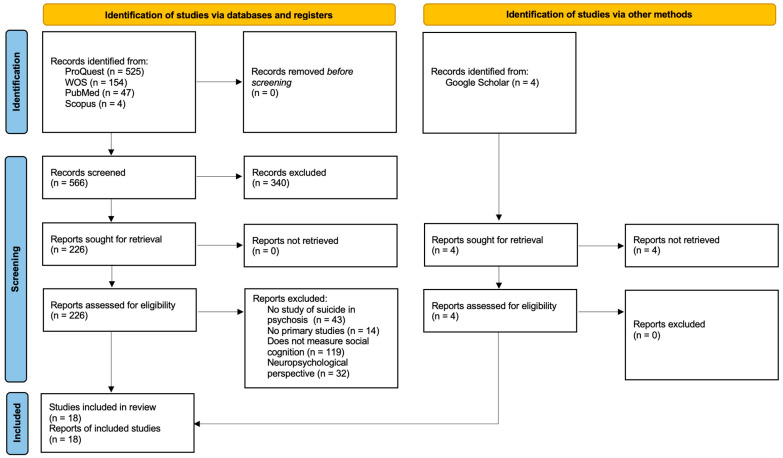
PRISMA 2020 flow diagram of studies on social cognition in psychosis and suicide behavior published between 2009 and 2024.

Initially, 730 articles were identified from the databases and four additional records from Google Scholar. All references were imported into Mendeley to remove duplicates, resulting in 566 unique publications. This process is illustrated in Figure 1, which presents the different phases followed during the two screening stages, culminating in a total of 18 articles included in the final qualitative synthesis.

### 2.4. Selection Process

To determine study eligibility, one researcher (MCH) screened the abstracts of the selected papers. The full text of potentially eligible studies were then assessed by the same reviewer (MCH). A double eligibility check was not performed. The literature search yielded 566 papers, of which 18 met the inclusion criteria (Figure 1). Only original articles were included.

### 2.5. Data Collection Process

One researcher (MCH) independently collected data from all reports. No automation tools were used during this process.

### 2.6. Data Items

Once the studies had been selected, a standardized form was used to extract the following data: authors, publication date, study design (longitudinal or cross-sectional), sample size, age, sex, diagnostic condition, and the psychological instruments used to assess both suicidal behavior and social cognition. Quality assessment of the included studies was performed using a tool for longitudinal and cross-sectional observational studies developed by the NIH (2014). This tool evaluates key concepts relevant to assessing the internal validity of each selected studies. Higher methodological quality was observed in the longitudinal studies (Canal-Rivero et al., 2017; Cuesta et al., 2022; Depp et al., 2018; Dickhoff et al., 2021; Parrish et al., 2024; Wastler et al., 2022), while the studies by Rocca et al. (2016), and Abdo et al. (2021) were most likely to be biased. A double checking of the quality assessment was not performed. A summary of each study is provided in Table S1. 

### 2.7. Study Risk of Bias Assessment

Risk of bias assessment was not conducted, as meta-analysis was not performed in this review. Conducting a meta-analysis was not an objective of this work due to the diversity of variables studied and the heterogeneity of the measurement instruments used across the included studies, which made it difficult to perform the corresponding statistical analyses.

### 2.8. Effect Measures

Due to the heterogeneity of the variables studied and the measurement instruments used across the included studies, no effect measures were selected or extracted in this review.

### 2.9. Synthesis Methods

The study characteristics, assessment measures, results and quality ratings were tabulated and are summarized in Table 1. Additionally, the measurement instruments used to assess social cognition, due to their variety, are presented in Table 2. No meta-analysis was performed in this review, as explained in Section 2.7.

### 2.10. Reporting Bias Assessment

As explained in Section 2.7, risk of bias assessment was not conducted because a meta-analysis was not performed in this review.

### 2.11. Certainty Assessment

The certainty in the body of evidence for outcomes in this review was assessed using the OCEBM Levels of Evidence Working Group (n.d.).

## 3. Results

Eighteen articles were included in this systematic review (Figure 1). The results and conclusions of the original studies and reviews are summarized in Table 1, highlighting the main findings of each work. Studies that identified important trends, such as effect sizes, were also included, as the limited number of studies and the controversial nature of the topic make these findings potentially valuable. Some studies performed regression analysis to predict suicidal behavior based on the variables examined or to analyze possible mediating variables; these variables are also described in the corresponding section.

### 3.1. Description of the Studies

Among the selected studies, 6 were longitudinal, involving repeated assessments over time, while 12 were cross-sectional, with a single measurement point. Cross-sectional studies, which account for 66.6% of the total number of studies, are less able to establish cause–effect relationships compared to longitudinal designs.

Regarding assessment instruments, numerous tools are available to assess suicidal behavior and social cognition; however, there is no consensus on the most appropriate tests for assessing these variables in schizophrenia (Gil-Sanz et al., 2019).

First, clinical interview is the most commonly used method to assess suicide, being employed on seven occasions to record the number of previous suicide attempts, quantitatively or dichotomously (Yes/No); the Schedule for Clinical Assessment in Neuropsychiatry (SCAN) is employed in similar circumstances (Vazquez-Barquero et al., 1994; Wing et al., 1990). The Columbia Suicide Severity Rating Scale (C-SSRS) (Al-Halabí et al., 2016; Posner et al., 2011) has also been widely used (on five occasions) to assess both suicide ideation and suicide attempt, as has The Suicide Probability Scale (SPS) (Cull & Gill, 1982). Other instruments, such as the Beck Depression Inventory (BDI-II) (Beck et al., 1961; Sanz et al., 2003), the Camberwell Questionnaire for Assessment of Needs (CANSAS) (Phelan et al., 1995; Rosales et al., 2002), the Modified Scale for Suicidal Ideation (MSSI) (Miller et al., 1986), and the Calgary Depression Scale (CDSS) (Addington et al., 1993; Sarró et al., 2004) are primarily used to assess suicidal ideation.

On the other hand, social cognition was assessed using 16 different instruments (Table 2). For theory of mind, the most widely used tools are the hinting task (Corcoran et al., 1995; Gil et al., 2012) and the Interpersonal Reactivity Index (IRI) (Davis, 1983; Pérez-Albéniz et al., 2003). For emotional processing, the Mayer–Salovey–Caruso Emotional Intelligence Test (MSCEIT) (Mayer et al., 2002; Extremera et al., 2006) and the Penn Emotion Recognition task (ER-40) (Kohler et al., 2003) are the most commonly used tools. For attributional style, the Ambiguous Intentions and Hostility Questionnaire (AIHQ) (Combs et al., 2007) and the Internal, Personal, and Situational Attributions Questionnaire (IPSAQ) (Kinderman & Bentall, 1996) are most common. Finally, the most commonly used tool for social perception is the Trustworthiness Task (Adolphs et al., 1998).

### 3.2. Sample

In all 18 original studies, there were 4511 participants. Sample sizes ranged from 21 to 809 participants, and seven studies included less than 100 participants. Sixteen of the eighteen studies involved an adult population aged 27–59 years, and two studies included a young adult population aged 23–26.2 years. Regarding sex distribution, one study included only males and another only females; in 12 studies, the majority of participants were male; in three studies, the majority were female; and one did not report on this information.

Regarding the diagnosis of the participants within the psychosis spectrum, only 50% of the studies considered this variable; the remaining studies did not apply consistent diagnostic classification, making it difficult to compare figures across variables. However, most samples consisted of individuals diagnosed with schizophrenia and schizoaffective disorder. Six studies included participants with bipolar disorder with psychotic symptoms, and four included individuals with major depressive disorder with psychotic symptoms. According to the DSM-5, these diagnosis are not classified within the psychosis spectrum, despite the presence of psychotic symptoms. Therefore, for comparison purposes, only the percentages for schizophrenic are reported. Using this approach, we found that in 72.2% of the studies, more than 50% of the sample was diagnosed with schizophrenia.

Regarding suicidal behavior, 12 of the studies assessed this variable as previous suicide attempts, most often recorded retrospectively through clinical interviews. The percentage of participants with a history of suicide attempts varied widely, ranging from 11.3% to 64.6%, with an average of approximately 35.5%. Suicidal ideation was evaluated in ten studies, with an average prevalence of 31.9%. Chapman et al. (2015) conducted a meta-analysis that demonstrated a strong association between suicidal ideation and suicidal behavior in psychotic disorders.

### 3.3. Emotional Processing

Emotional processing was the most studied social cognition domain, examined in 61.1% (11) of the articles and assessed by eight different instruments. Depp et al. (2018), using the BLERT emotion identification task (Bryson et al., 1997), found that participants with current suicidal ideation were more accurate in rating negative affect (*p* = 0.042, d = 0.441). This finding agrees with Dickhoff et al. (2021), who also used the DFAR (Van’t Wout et al., 2004) and obtained better performance in face detection, especially for fear expressions, in participants with a history of suicide attempts or current suicidal ideation (F (1,586) = 4.1, *p* = 0.04). Thus, we found a tendency toward better emotional processing for emotion recognition, particularly for negative emotions. 

However, Harenski et al. (2017) found worse emotion identification using their ad hoc test of empathic accuracy in participants with a history of suicidal behavior, although this study only included a prison population (*p* = 0.032). Parrish et al. (2024) and Villa et al. (2018) found no significant relationship between emotion recognition and suicidal behavior but observed a misattribution of anger or fear to neutral faces (*p* < 0.01, d = 0.141). They also found an over-attribution bias for threat, which was related to perceived burden to others (t = 4.72, *p* < 0.001), reduced social motivation (t = 3.33, *p* < 0.001), and increased social avoidance (t = 3.70, *p* < 0.001). These constructs are related to suicide and may therefore indirectly contribute to suicidal behavior.

Comparelli et al. (2018) found that greater severity of suicidal ideation was associated with lower performance on the MSCEIT (r = 0.329, *p* < 0.05) (Mayer et al., 2002; Extremera et al., 2006), a test that assesses not only emotion recognition but also the ability to identify, understand and manage emotions. Furthermore, they indicated that this deficit may serve as a potential predictor of suicidal ideation (*p* = 0.015).

The evidence for emotional processing is supported by a relatively large number of studies (*n* = 11), with generally consistent findings regarding the association between emotional recognition—particularly of negative emotions—and suicidal behavior. However, the certainty of this evidence is limited by methodological heterogeneity, variability in assessment tools, and the predominance of cross-sectional designs, as well as some conflicting results. Overall, confidence in evidence is moderate, downgraded due to these limitations but strengthened by the number of studies and the overall consistency of the main findings.

### 3.4. Theory of Mind

Theory of mind was the most studied concept along with emotional processing, investigated in 50% (9) of the studies using five different assessment instruments. Dickhoff et al. (2021) found better performance in the hinting task (Corcoran et al., 1995; Gil et al., 2012) among participants with previous suicide attempts or current suicidal ideation (F (1,644) = 4.4, *p* = 0.04). Furthermore, participants with both conditions (suicide attempts and current suicidal ideation) performed better than those with only one condition. This finding was supported by Sastre-Buades et al. (2023), who observed the same trend with a small mean effect size (t = 2.04, d = 0.403), although their results did not reach statistical significance. 

In contrast, using the FBT task (Frith & Corcoran, 1996), studies by Canal-Rivero et al. (2017) and Duñó et al. (2009) found worse performance in participants with a history of suicidal behavior for second-order tasks (X2 = 6.27, *p* = 0.01; X2 = 5.223, *p* = 0.022). The first study also reported worse performance for first-order tasks (X2 = 3.95, *p* = 0.04). Additionally, they identified both first-order (OR = 4.26, CI = 1.05–17.31, *p* = 0.04) and second-order (OR = 4.02, CI = 1.18–13.62, *p* < 0.05) task performance as potential predictors of increased suicidal behavior at 12-month follow-up.

On the other hand, using the IRI index (Davis, 1983; Pérez-Albéniz et al., 2003), we found significant results (*p* = 0.002) related to personal distress (F = 5.045, *p* = 0.020; t = −3.166, *p* = 0.002), defined as the anxiety and discomfort produced by observing negative experiences of others and considered part of affective empathy. Significant results were also found for fantasy (F = 10.445, *p* = 0.001), which represents the tendency to identify with others and is part of cognitive empathy) (Liu et al., 2023; Wang et al., 2020; Zhu & Zhang, 2024). 

Finally, Abdo et al. (2021) found a deficit in the affective component of theory of mind as assessed by the RMET (Baron-Cohen et al., 2001; Fernández-Abascal et al., 2013), which correlated negatively with the hopelessness subdomain of suicidal risk (R = − 0.526, *p* = 0.017), although not with overall suicidal risk. In addition, Liu et al. (2023) and Wang et al. (2020) identified personal distress score, a component of affective empathy, as a predictor of suicide attempts and suicidal ideation (OR = 1.092, CI = 1.013–1.177, *p* = 0.022; OR = 1.076, CI = 1.013–1.142, *p* = 0.017).

Therefore, following Jarvis et al. (2024) and their division between cognitive and affective theory of mind, we observe that the method of assessment can significantly influence results for the cognitive component, as different findings were obtained depending on the instrument used (Canal-Rivero et al., 2017; Dickhoff et al., 2021; Duñó et al., 2009; Sastre-Buades et al., 2023). Concerning the affective component, the results are similar: all three studies that assessed this domain found that higher scores were related to suicidal behavior, although Abdo et al. (2021) found the opposite pattern with hopelessness related to suicide but not to suicidal behavior itself.

Theory of mind was investigated in nine studies using various assessment instruments, with mixed and sometimes conflicting results depending on the specific aspect and tool evaluated (cognitive vs. affective theory of mind). While some studies found better performance or higher empathy in individuals with suicidal behavior, others reported worse performance or no significant association, and findings often varied depending on the instrument and outcome. The certainty of the evidence is limited by methodological heterogeneity, inconsistency across studies, and the predominance of cross-sectional designs. Confidence in evidence is low to moderate, downgraded due to these limitations but strengthened by the number of studies and the exploration of both cognitive and affective components.

### 3.5. Attributional Style

Attributional style was studied in 16.6% (3) of the studies, using two different assessment instruments. Statistically significant results were found only with the AIHQ questionnaire (Combs et al., 2007), which showed a specific tendency to blame others in participants with suicidal ideation (*p* = 0.006, d = 0.599) (Depp et al., 2018). However, in the study by Chalker et al. (2022), this was observed only in the sample that had experienced emotional (t = 2.75, *p* = 0.007) or physical (t = 2.39, *p* = 0.019) abuse. Furthermore, Depp et al. (2018) identified a hyper attribution of blame to others, especially in participants with recurrent suicidal ideation present at two time points, as a predictor of greater future suicidal ideation (OR = 1.203, CI = 1.045–1.386, *p* = 0.010). In the study by Sastre-Buades et al. (2023), no statistically significant results were found, but a small effect size (t = 2.07, d = 0.482) indicated a tendency to make internal attributions for more negative events, similar to that associated with depressive disorders, i.e., a lower externalizing bias in participants with a history of suicide attempt. 

The evidence for attributional style is based on three studies using two different assessment instruments. Significant results were mainly found with the AIHQ, indicating a tendency to blame others in participants with suicidal ideation and a lower externalizing bias in those with a history of suicide attempts. However, the strength of the evidence is limited by the small number of studies, variability in assessment tools, small to moderate effect sizes, predominance of cross-sectional designs, and lack of replication. Confidence in evidence is low, downgraded due to these limitations.

### 3.6. Social Perception

Social perception is the least studied domain within social cognition, being assessed in only one study, by Depp et al. (2018). In this study, participants with higher current ideation demonstrated greater normative unreliability in judging untrustworthy faces, as measured by the Trustworthiness Task (*p* = 0.042, d = 0.39) (Adolphs et al., 1998). The strength of evidence is limited, as only one study at level 3 evidence is available, with no replication, a small effect size, and possible methodological limitations. Therefore, confidence in the evidence is currently low.

### 3.7. Additional Analyses

Some of the 18 studies included in the final analysis examined additional variables that might mediate the relationship between social cognition and suicidal behavior in psychosis. Liu et al. (2023) noted that scores on positive and negative syndrome and general psychopathology, as assessed by the Positive and Negative Syndrome Scale for Schizophrenia (PANSS) (Kay et al., 1987; Peralta & Cuesta, 1994), might mediate the relationship between personal distress, as part of theory of mind, and suicidal ideation. 

Something similar was found in Dickhoff et al. (2021), who also noted that the PANSS score (Kay et al., 1987; Peralta & Cuesta, 1994) could mediate between emotion recognition as part of emotional processing and the hinting task as part of theory of mind, and current ideation or past suicidal intent. Depp et al. (2018) identified another factor, the depression score, as a possible mediator in the relationship between negative biases —such as the tendency to blame others, greater unreliability in unreliable faces, and greater accuracy in rating negative affect— and suicidal ideation. Zhu and Zhang (2024) mentioned cognitive performance measured by the Mini-Mental State Examination (MMSE) (Folstein et al., 1983; Lobo et al., 1999) and gender as possible mediating factors in the relationship between empathy and suicidal ideation, finding greater empathy in men with cognitive dysfunction. 

Finally, Chalker et al. (2022) identified emotional trauma, as measured by the Childhood Trauma Questionnaire (CTQ) (Bernstein et al., 1998; Hernandez et al., 2013), as a mediator between the tendency to blame others, as part of attributional style, and suicidal behavior.

## 4. Discussion

In this systematic review, we identified differences across social cognition domains that are related to increased suicidal behavior in individuals with psychotic disorders. Individuals with psychotic disorder and suicidal behavior showed better emotional processing in basic emotional recognition tasks, but poorer in more complex tasks, as well as greater empathy within the affective theory of mind. Cognitive biases, such as a tendency to blame others as part of attributional style, and greater distrust as part of social perception, were also found in patients with higher suicidal behavior.

Within emotional processing, we found complementary results. In studies where basic emotions recognition was assessed, better performance was observed, especially for negative emotions (Depp et al., 2018; Dickhoff et al., 2021). However, Harenski et al. (2017) report the opposite, indicating inconsistencies in the literature. Parrish et al. (2024) and Villa et al. (2018) observed an over-attribution of threat bias, which could be indirectly related to increased suicidal behavior, as it is associated with perceptions of being a burden to others, reduced social motivation, and increased social avoidance. Comparelli et al. (2018) found that poorer performance was related to greater severity of suicidal ideation; however, their measurement instrument was much more complex and extended beyond emotion recognition. Thus, individuals may perform better on basic affect ratings but worse in more complex tasks. These findings may be due to greater reactivity to and accuracy of negative stimuli as part of negative bias, and these affect recognition biases could contribute to increased perceptions of isolation and lack of support, concepts related to suicidal behavior (Depp et al., 2018). Additionally, it appears that individuals with higher suicidal behavior demonstrate a greater ability to detect negative social cues and exhibit a threat over-attribution bias, where negative emotions are misattributed to neutral faces (Dickhoff et al., 2021; Sastre-Buades et al., 2023).

We found the greatest discrepancies between studies regarding the theory of mind domain. Within the cognitive component, better performance was observed in the interpretation of hints during social interactions, particularly among individuals with more severe suicidal behavior (Dickhoff et al., 2021; Sastre-Buades et al., 2023). However, when theory of mind was assessed using tasks that involve both simple and a complex forms of interpersonal problem-solving, such as predicting false beliefs, the opposite pattern emerged (Canal-Rivero et al., 2017; Duñó et al., 2009). In contrast, when the affective component of theory of mind was examined, findings were more consistent across studies, although not entirely uniform. A higher score was observed, particularly in personal distress. which is a component of affective empathy defined as the emotional response of an individual who observes the affective state of others (Liu et al., 2023; Wang et al., 2020; Zhu & Zhang, 2024). However, Abdo et al. (2021), using a different measurement instrument that assess empathic accuracy, found poorer performance, although this was related only to hopelessness and not to suicidal behavior itself. Overall, our findings suggest that participants with higher suicidal behavior tend to show higher empathy, which may be explained by increased anxiety and discomfort when observing the negative experiences of others, or by heightened anxiety in stressful interpersonal environments. This heightened emotional reactivity may negatively affect their perception of support, thereby contributing to increased suicidal behavior (Liu et al., 2023; Wang et al., 2020).

For attributional style and social perception, we found few studies reporting statistically significant results. Greater unreliability and a tendency to blame others were observed in patients with higher suicidal behavior (Chalker et al., 2022; Depp et al., 2018). Depp et al. (2018) hypothesized that a negative attribution bias is linked to suicidal ideation. Additionally, Sastre-Buades et al. (2023) observed a tendency to a very low externalizing bias in individuals with a history of suicide attempts.

In our approach to identifying possible variables that mediate this relationship, we found that psychopathology (including positive and negative syndrome, depression, and emotional trauma), cognitive performance, and gender could be involved. However, further studies are needed to clarify these associations (Chalker et al., 2022; Depp et al., 2018; Dickhoff et al., 2021; Liu et al., 2023; Zhu & Zhang, 2024).

Several strengths and limitations emerged throughout this systematic review. This review aimed to clarify whether social cognition is a risk factor for a major cause of mortality in these patients. Furthermore, differentiating between subdomains allows for better clinical application when considering this factor in treatment. Several limitations also emerged in this review. The main limitation is the difficulty in defining and measuring variables with no clear consensus (Gil-Sanz et al., 2019; Green et al., 2008; Pinkham, 2014) and the predominance of cross-sectional methodologies, with more than 60% of studies employing this design. There is also a tendency for research on social cognition to focus primarily on emotional processing and theory of mind (Healey et al., 2016).

Further research on social cognition in psychosis, and specifically on whether there is a determinant relationship with suicidal behavior, is important to clarify whether social cognition constitutes a risk factor for one of the main causes of mortality in these population, and to inform clinical applications focused on optimizing and individualizing treatment. More longitudinal studies and more comprehensive analyses assessing all domains, as well as possible mediating variables in this relationship are potential future lines of research.

## 5. Conclusions

Better emotional processing in simpler tasks, such as emotional recognition but poorer performance in more complex tasks, as well as greater empathy within the affective theory of mind, appear to be associated with increased suicidal behavior. Biases, such as a tendency to blame others as part of attributional style and greater distrust as part of social perception, are also observed in patients with higher suicidal behavior. Further studies are needed to clarify the relationship with the cognitive theory of mind, to integrate the four subdomains in more longitudinal studies, and to investigate the variables that influence these relationships.

## Figures and Tables

**Table 1:** Summary of the studies included for the systematic review about the relationship between social cognition and suicidal behavior in psychosis.

Author and Year	Design and Sample	Measure of Suicidal Behavior	Social Cognition Assessment	Results	Quality Assessment by the NIH
Abdo et al. (2021)	Transversal *n* = 21; Mean age = 30.1; F = 100%; Schizophrenia = 100%	SPS	ToM: RMET	Suicidal ideation (hopelessness subdomain): lower affective theory of mind.	Fair
Canal-Rivero et al. (2017)	Longitudinal (12 months) *n* = 65; Mean age = 26.2; M = 67.7%; F = 32.3%; Schizophrenia = 70.8%	SCAN	ToM: FBT, Hinting task	History of suicide attempt: more errors in first- and second-order false belief tasks. Predictor: deficit in first-order tasks.	Good
Chalker et al. (2022)	Transversal *n* = 96; Mean age = 43.8; M = 44.8%; F = 55.2%; Schizophrenia = 38.5%	C-SSRS	AS: AIHQ	Suicidal ideation: tendency to blame others in individuals with history of emotional or physical abuse.	Fair
Comparelli et al. (2018)	Transversal *n* = 86; Mean age = 36.3; M = 74%; F = 26%	C-SSRS	EP: MSCEIT	Suicidal ideation: greater severity related to impairment in social cognition. Predictor: deficit in social cognition.	Fair
Cuesta et al. (2022)	Longitudinal (20 years) *n* = 172; Mean age = 48.1; M = 51.7%; F = 48.3%; Schizophrenia = 43.6%	Clinical interview	EP: MSCEIT	No statistically significant relationship.	Good
Depp et al. (2018)	Longitudinal (2 weeks) *n* = 179; Mean age = 42.1; M = 65.4%; F = 34.6%; Schizophrenia = 53.1%	BDI-2	EP: BLERT, ER-40 SP: Trustworthiness Task AS: AIHQ	Suicidal ideation: greater tendency to blame others, greater unreliability in unreliable faces, and greater accuracy in rating negative affect. Predictor: attribution of blame to others, especially in persistent ideation.	Good
Dickhoff et al. (2021)	Longitudinal (3 years) *n* = 715; Mean age = 27.2; M = 72.4%; F = 27.6%; Schizophrenia = 70.4%	Clinical interview, CANSAS	EP: DFAR, BFRT ToM: Hinting task	Suicide ideation/attempt: better performance in face detection, especially for fear emotion, and better performance in hinting task in participants with suicidal ideation and attempt.	Good
Duñó et al. (2009)	Transversal *n* = 57; Mean age = 31.2; M = 70.1%; F = 29.9%; Schizophrenia = 100%	Clinical interview	ToM: FBT	History of suicide attempt: worse performance in second-order false belief task. Predictor: second-order task.	Fair
Harenski et al. (2017)	Transversal *n* = 41; Mean age = 39.7; M = 100%; Schizophrenia = 46.3% Prison population	C-SSRS	EP: Empathic accuracy task	History of suicide attempt: lower empathic accuracy. Predictor: empathic accuracy.	Fair
Liu et al. (2023)	Transversal *n* = 301; Mean age = 33.9; M = 60.8%; F = 39.2%; Schizophrenia = 100%	BDI	ToM: IRI	Suicidal ideation: greater personal distress (affective empathy). Predictor: personal distress (affective empathy).	Fair
Parrish et al. (2024)	Longitudinal (10 days) *n* = 273; Mean age = 41.7; M = 62.6%; F = 37.4%; Schizophrenia = 26%	C-SSRS	EP: ER-40	No statistically significant relationship between ER-40 and suicide risk, but greater over attribution of threat related to interpersonal constructs related to suicidal behavior.	Good
Rocca et al. (2016)	Transversal *n* = 809; Mean age = 40.1; Schizophrenia = 100%	Clinical interview	EP: MSCEIT, FEIT ToM: TASIT	No statistically significant relationship.	Poor
Sastre-Buades et al. (2023)	Transversal *n* = 190; Mean age = 27.9; M = 67.2%; F = 32.8%; Schizophrenia = 38.4%	Clinical interview	EP: Baron Cohen’s Face Test (CBC) ToM: Hinting task AS: IPSAQ	No statistically significant relationship. Small mean effect size for better performance in hinting tasks and very low externalizing bias in patients with suicide attemp history.	Fair
Villa et al. (2018)	Transversal *n* = 101; Mean age = 49.9; M = 45.5%; F = 54.5%; Schizophrenia + Schizoaffective = 74.3%	C-SSRS, MSSI	EP: ER-40	No statistically significant relationship. Tendency in patients with history of suicide to misattribute threat to neutral stimuli.	Fair
Wang et al. (2020)	Transversal *n* = 627; Mean age = 47.7; M = 63%; F = 37%; Schizophrenia = 100%	Clinical interview	ToM: IRI	Suicide attempt history: greater total empathy, fantasy (cognitive empathy) and personal distress (affective empathy). Predictor: personal distress (affective empathy).	Fair
Wastler et al. (2022)	Longitudinal (6 months) *n* = 65; Mean age = 23; M = 70.8%; F = 29.2%; Schizophrenia spectrum = 60%	CDSS	EP: MSCEIT	No statistically significant relationship	Good
Yin et al. (2020)	Transversal *n* = 159; Mean age = 27.1; M = 47.8%; F = 52.2%; Schizophrenia = 100%	Clinical interview	EP: MSCEIT	No statistically significant relationship	Fair
Zhu and Zhang (2024)	Transversal *n* = 554; Mean age = 46.4; M = 68.4%; F = 31.6%; Schizophrenia = 100%	Clinical interview; BDI	ToM: IRI	Suicidal ideation: greater personal anguish (affective empathy)	Fair

Note: F: female; M: male; ToM: theory of mind; AS: attributional style; SP: social perception; EP: emotional processing; NIH: National Institutes of Health.

**Table 2:** Summary of instruments for measuring different domains of social cognition.

	Description	Authors
Reading the Mind in the Eyes Test (RMET)	ToM. Recognition of emotions through photographs of the eyes; the participant must choose the word that most represents the emotion.	(Baron-Cohen et al., 2001; Fernández-Abascal et al., 2013)
The Awareness of Social Inference Test (TASIT)	ToM. Audiovisual tool integrating gestures, prosody, facial expression, and context to interpret meanings, emotions, beliefs and intentions.	(McDonald et al., 2006)
Hinting Task	ToM. Stories describing a social interaction with a hint that the participant must interpret.	(Corcoran et al., 1995; Gil et al., 2012)
False Belief Test (FBT)	ToM. First order task: predict the behavior of a character guided by a false belief. Second order task: predict the false belief that one character has about another character’s belief.	(Frith & Corcoran, 1996)
Interpersonal Reactivity Index (IRI)	ToM. Divided into cognitive empathy (understanding another’s perspective) and affective empathy (emotional response to another’s affective state).	(Davis, 1983; Pérez-Albéniz et al., 2003)
Internal, Personal and Situational Attributions Questionnaire (IPSAQ)	AS. Ability to discriminate between external and internal attributions, both personal and situational, through hypothetical scenarios.	(Kinderman & Bentall, 1996)
Ambiguous Intentions and Hostility Questionnaire (AIHQ)	AS. Analyzes the tendency to overattribute negative intentions to others using vignettes of social situations, asking about the intentions of the characters and the participant’s own likely response,	(Combs et al., 2007)
Trustworthiness Task (TT)	SP. Ability to make complex social judgments of trustworthiness from facial photographs.	(Adolphs et al., 1998)
Mayer-Salovey-Caruso Emotional Intelligence Test (MSCEIT)	EP. Tests assessing emotional perception, emotional facilitation, emotional understanding, and emotional management as skills for handling emotional problems.	(Mayer et al., 2002; Extremera et al., 2006)
The Bell Lysaker Emotion Recognition Task (BLERT)	EP. Identification of emotion through the intonation used by an actor interpreting emotions in videos.	(Bryson et al., 1997)
Penn Emotion Recognition task (ER-40)	EP. Recognition of basic emotions through facial photographs.	(Kohler et al., 2003)
Degraded Facial Affect Recognition Task (DFAR)	EP. Computer images of people displaying basic emotions, shown with increasing intensity to raise difficulty.	(Van’t Wout et al., 2004)
Baron Cohen’s Face Test (CBC)	EP. Photographs of an actress representing an emotion; the participant must choose the emotion that best fits.	(Baron-Cohen et al., 1997; Huerta-Ramos et al., 2021)
The Benton Facial Recognition Task (BFRT)	EP. Assesses face memory by showing a series of face cards, which the participant must identify among several photographs.	(Benton et al., 1983)
Facial Emotion Identification Test (FEIT)	EP. Identification of basic emotions through facial photographs.	(Kerr & Neale, 1993)

Note: ToM: Theory of mind; AS: attributional style; SP: social perception; EP: emotional processing.

## Data Availability

No new data were created or analyzed in this study. Data sharing is not applicable to this article.

## Supplementary Materials

The following supporting information can be downloaded at: https://www.mdpi.com/article/10.3390/bs15060759/s1, Table S1: Quality assessment of studies using a tool designed by the NIH (2014) for longitudinal and cross-sectional observational designs.

## References

[B1-behavsci-15-00759] Abdo M. M. M., Mohamed A. S., Hammed M. A. E., Hashem R. E., El Nagar Z. M. (2021). Affective theory of the mind and suicide in women with borderline personality disorder and schizophrenia: A comparative study. Middle East Current Psychiatry.

[B2-behavsci-15-00759] Addington D., Addington J., Maticka-Tyndale E. (1993). Assessing depression in schizophrenia: The Calgary depression scale. The British Journal of Psychiatry.

[B3-behavsci-15-00759] Adolphs R., Tranel D., Damasio A. R. (1998). The human amygdala in social judgment. Nature.

[B4-behavsci-15-00759] Al-Halabí S., Sáiz P. A., Burón P., Garrido M., Benabarre A., Jiménez E., Cervilla J., Navarrete M. I., Díaz-Mesa E. M., García-Álvarez L., Muñiz J. (2016). Validation of a Spanish version of the Columbia-suicide severity rating scale (C-SSRS). Revista de Psiquiatría y Salud Mental.

[B5-behavsci-15-00759] Baron-Cohen S., Wheelwright S., Hill J., Raste Y., Plumb I. (2001). The “reading the mind in the eyes” test revised version: A study with normal adults, and adults with Asperger syndrome or high-functioning autism. The Journal of Child Psychology and Psychiatry and Allied Disciplines.

[B6-behavsci-15-00759] Baron-Cohen S., Wheelwright S., Jolliffe A. T. (1997). Is there a” language of the eyes”? Evidence from normal adults, and adults with autism or Asperger syndrome. Visual Cognition.

[B7-behavsci-15-00759] Beck A. T., Ward C. H., Mendelson M., Mock J., Erbaugh J. (1961). An inventory for measuring depression. Archives of General Psychiatry.

[B8-behavsci-15-00759] Benton A., Sivan P. A., Hamsher K., Varney D., Spreen O. (1983). Facial recognition: Stimulus and multiple choice pictures: Contributions to neuropsychological assessment.

[B9-behavsci-15-00759] Bernstein D. P., Fink L., Handelsman L., Foote J. (1998). Childhood trauma questionnaire. Assessment of family violence: A handbook for researchers and practitioners.

[B10-behavsci-15-00759] Bora E., Pantelis C. (2013). Theory of mind impairments in first-episode psychosis, individuals at ultra-high risk for psychosis and in first-degree relatives of schizophrenia: Systematic review and meta-analysis. Schizophrenia Research.

[B11-behavsci-15-00759] Bryson G., Bell M., Lysaker P. (1997). Affect recognition in schizophrenia: A function of global impairment or a specific cognitive deficit. Psychiatry Research.

[B12-behavsci-15-00759] Canal-Rivero M., Lopez-Moriñigo J. D., Barrigón M. L., Perona-Garcelán S., Jimenez-Casado C., David A. S., Obiols-Llandrich J. E., Ruiz-Veguilla M. (2017). The role of premorbid personality and social cognition in suicidal behaviour in first-episode psychosis: A one-year follow-up study. Psychiatry Research.

[B13-behavsci-15-00759] Cassidy R. M., Yang F., Kapczinski F., Passos I. C. (2018). Risk factors for suicidality in patients with schizophrenia: A systematic review, meta-analysis, and meta-regression of 96 studies. Schizophrenia Bulletin.

[B14-behavsci-15-00759] Chalker S. A., Parrish E. M., Cano M., Kelsven S., Moore R. C., Granholm E., Pinkham A., Harvey P. D., Depp C. A. (2022). Childhood trauma associations with the interpersonal psychological theory of suicide and social cognitive biases in psychotic disorders. The Journal of Nervous and Mental Disease.

[B15-behavsci-15-00759] Chapman C. L., Mullin K., Ryan C. J., Kuffel A., Nielssen O., Large M. M. (2015). Meta-analysis of the association between suicidal ideation and later suicide among patients with either a schizophrenia spectrum psychosis or a mood disorder. Acta Psychiatrica Scandinavica.

[B16-behavsci-15-00759] Combs D. R., Penn D. L., Wicher M., Waldheter E. (2007). The Ambiguous Intentions Hostility Questionnaire (AIHQ): A new measure for evaluating hostile social-cognitive biases in paranoia. Cognitive Neuropsychiatry.

[B17-behavsci-15-00759] Comparelli A., Corigliano V., Lamis D. A., De Carolis A., Stampatore L., De Pisa E., Girardi P., Pompili M. (2018). Positive symptoms and social cognition impairment affect severity of suicidal ideation in schizophrenia. Schizophrenia Research.

[B18-behavsci-15-00759] Comparelli A., Corigliano V., Montalbani B., Nardella A., De Carolis A., Stampatore L., Bargagna P., Forcina F., Lamis D., Pompili M. (2022). Building a neurocognitive profile of suicidal risk in severe mental disorders. BMC Psychiatry.

[B19-behavsci-15-00759] Corcoran R., Mercer G., Frith C. D. (1995). Schizophrenia, symptomatology and social inference: Investigating “theory of mind” in people with schizophrenia. Schizophrenia Research.

[B20-behavsci-15-00759] Correll C. U., Solmi M., Croatto G., Schneider L. K., Rohani-Montez S. C., Fairley L., Smith N., Bitter I., Gorwood P., Taipale H., Tiihonen J. (2022). Mortality in people with schizophrenia: A systematic review and meta-analysis of relative risk and aggravating or attenuating factors. World Psychiatry.

[B21-behavsci-15-00759] Cuesta M. J., Sánchez-Torres A. M., Moreno-Izco L., de Jalón E. G., Gil-Berrozpe G. J., Zarzuela A., Peralta V., Ballesteros A., Fañanás L., Hernández R., Janda L. (2022). Neurocognitive correlates of the varied domains of outcomes at 20 year follow-up of first-episode psychosis. Psychiatry Research.

[B22-behavsci-15-00759] Cull J. G., Gill W. S. (1982). Suicide probability scale (SPS).

[B23-behavsci-15-00759] Davis M. H. (1983). Measuring individual differences in empathy: Evidence for a multidimensional approach. Journal of Personality and Social Psychology.

[B24-behavsci-15-00759] Depp C. A., Villa J., Schembari B. C., Harvey P. D., Pinkham A. (2018). Social cognition and short-term prediction of suicidal ideation in schizophrenia. Psychiatry Research.

[B25-behavsci-15-00759] Dickhoff J., Opmeer E. M., Heering H. D., Bruggeman R., van Amelsvoort T., Bartels-Velthuis A. A., Cahn W., de Haan L., Schirmbeck F., Simons C., van Os J., Aleman A., van Tol M. J. (2021). Relationship between social cognition, general cognition, and risk for suicide in individuals with a psychotic disorder. Schizophrenia Research.

[B26-behavsci-15-00759] Duñó R., Pousa E., Miguélez M., Montalvo I., Suarez D., Tobeña A. (2009). Suicidality connected with mentalizing anomalies in schizophrenia: A study with stabilized outpatients. Annals of the New York Academy of Sciences.

[B27-behavsci-15-00759] Extremera N., Fernández-Berrocal P., Salovey P. (2006). Spanish version of the Mayer-Salovey-Caruso Emotional Intelligence Test (MSCEIT). Version 2.0: Reliabilities, age and gender differences. Psicothema.

[B28-behavsci-15-00759] Fernández-Abascal E. G., Cabello R., Fernández-Berrocal P., Baron-Cohen S. (2013). Test–retest reliability of the ‘reading the mind in the eyes’ test: A one-year follow-up study. Molecular Autism.

[B29-behavsci-15-00759] Folstein M. F., Robins L. N., Helzer J. E. (1983). The mini-mental state examination. Archives of General Psychiatry.

[B30-behavsci-15-00759] Fortuny J. J., Navarra-Ventura G., Fernández-Gonzalo S., Tomàs E. P., Armengol J. M. C., Vidal D. P., Vicente M. J. (2023). Social cognition in first-episode schizophrenia/schizoaffective disorder patients. Spanish Journal of Psychiatry and Mental Health.

[B31-behavsci-15-00759] Frith C. D., Corcoran R. (1996). Exploring ‘theory of mind’ in people with schizophrenia. Psychological Medicine.

[B32-behavsci-15-00759] Fu X. L., Qian Y., Jin X. H., Yu H., Wu H. R., Du L., Chen H., Shi Y. Q. (2023). Suicide rates among people with serious mental illness: A systematic review and meta-analysis. Psychological Medicine.

[B33-behavsci-15-00759] Gil D., Fernández-Modamio M., Bengochea R., Arrieta M. (2012). Adaptation of the Hinting Task theory of the mind test to Spanish. Revista de Psiquiatría y Salud Mental.

[B34-behavsci-15-00759] Gil-Sanz D., Bengochea-Seco R., Arrieta-Rodríguez M., Santacoloma-Cabero I., González-Fraile E. (2019). ¿Cómo se evalúa la cognición social en esquizofrenia en España?. Informaciones Psiquiátricas.

[B35-behavsci-15-00759] Green M. F., Penn D. L., Bentall R., Carpenter W., Gaebel W. T., Gur R. C., Kring A., Park S., Silverstein S., Heinssen R. (2008). Social cognition in schizophrenia: An NIMH workshop on definitions, assessment, and research opportunities. Schizophrenia Bulletin.

[B36-behavsci-15-00759] Harenski C. L., Brook M., Kosson D. S., Bustillo J. R., Harenski K. A., Caldwell M. F., Van Rybroek G. J., Koenigs M., Decety J., Thornton D. M., Calhoun V. D. (2017). Socio-neuro risk factors for suicidal behavior in criminal offenders with psychotic disorders. Social Cognitive and Affective Neuroscience.

[B37-behavsci-15-00759] Healey K. M., Bartholomeusz C. F., Penn D. L. (2016). Deficits in social cognition in first episode psychosis: A review of the literature. Clinical Psychology Review.

[B38-behavsci-15-00759] Hernandez A., Gallardo-Pujol D., Pereda N., Arntz A., Bernstein D. P., Gaviria A. M., Labad A., Valero J., Gutiérrez-Zotes J. A. (2013). Initial validation of the Spanish childhood trauma questionnaire-short form: Factor structure, reliability and association with parenting. Journal of Interpersonal Violence.

[B39-behavsci-15-00759] Hor K., Taylor M. (2010). Review: Suicide and schizophrenia: A systematic review of rates and risk factors. Journal of Psychopharmacology.

[B40-behavsci-15-00759] Huerta-Ramos E., Ferrer-Quintero M., Gómez-Benito J., González-Higueras F., Cuadras D., del Rey-Mejías Á. L., Usall J., Ochoa S. (2021). Translation and validation of Baron Cohen’s Face Test in a general population from Spain. Actas Españolas de Psiquiatría.

[B41-behavsci-15-00759] Instituto Nacional de Estadística (2023). Estadística de defunciones según la causa de muerte.

[B42-behavsci-15-00759] Jarvis A. L., Keage H. A., Wong S., Weightman M., Stephens R. G. (2024). Evidence for a multidimensional account of cognitive and affective theory of mind: A state-trace analysis. Memory & Cognition.

[B43-behavsci-15-00759] Kay S. R., Fiszbein A., Opler L. A. (1987). The positive and negative syndrome scale (PANSS) for schizophrenia. Schizophrenia Bulletin.

[B44-behavsci-15-00759] Keilp J. G., Beers S. R., Burke A. K., Melhem N. M., Oquendo M. A., Brent D. A., Mann J. J. (2014). Neuropsychological deficits in past suicide attempters with varying levels of depression severity. Psychological Medicine.

[B45-behavsci-15-00759] Keilp J. G., Gorlyn M., Russell M., Oquendo M. A., Burke A. K., Harkavy-Friedman J., Mann J. J. (2013). Neuropsychological function and suicidal behavior: Attention control, memory and executive dysfunction in suicide attempt. Psychological Medicine.

[B46-behavsci-15-00759] Kerr S. L., Neale J. M. (1993). Emotion perception in schizophrenia: Specific deficit or further evidence of generalized poor performance?. Journal of Abnormal Psychology.

[B47-behavsci-15-00759] Kinderman P., Bentall R. P. (1996). A new measure of causal locus: The internal, personal and situational attributions questionnaire. Personality and Individual Differences.

[B48-behavsci-15-00759] Kohler C. G., Turner T. H., Bilker W. B., Brensinger C. M., Siegel S. J., Kanes S. J., Gur R. E., Gur R. C. (2003). Facial emotion recognition in schizophrenia: Intensity effects and error pattern. American Journal of Psychiatry.

[B49-behavsci-15-00759] Kohler C. G., Walker J. B., Martin E. A., Healey K. M., Moberg P. J. (2010). Facial emotion perception in schizophrenia: A meta-analytic review. Schizophrenia Bulletin.

[B50-behavsci-15-00759] Lan X., Zhou Y., Zheng W., Zhan Y., Liu W., Wang C., Jiang M., Yu M., Zhang B., Ning Y. (2020). Association between cognition and suicidal ideation in patients with major depressive disorder: A longitudinal study. Journal of Affective Disorders.

[B51-behavsci-15-00759] Liu J., Zhao K., Zhou S., Hong L., Xu Y., Sun S., Tong S., Huang L., Liu J., Wang J., Li N. (2023). Suicidal ideation in Chinese adults with schizophrenia: Associations with neurocognitive function and empathy. BMC Psychiatry.

[B52-behavsci-15-00759] Lobo A., Saz P., Marcos G., Día J. L., de la Cámara C., Ventura T., Morales Asín F., Fernando Pascual L., Montañés J. Á., Aznar S. (1999). Revalidación y normalización del Mini-Examen Cognoscitivo (primera versión en castellano del Mini-Mental Status Examination) en la población general geriátrica. Medicine Clínica (Barc).

[B53-behavsci-15-00759] Lu L., Dong M., Zhang L., Zhu X. M., Ungvari G., Ng C., Wang G., Xiang Y. T. (2019). Prevalence of suicide attempts in individuals with schizophrenia: A meta-analysis of observational studies. Epidemiology and Psychiatric Sciences.

[B55-behavsci-15-00759] Martín M. C., Secades R., López-Goñi J. J., Tirapu J. (2017). Empatía, cognición social y calidad de vida subjetiva en esquizofrenia. Anales del Sistema Sanitario de Navarra.

[B56-behavsci-15-00759] Mayer J. D., Salovey P., Caruso D. (2002). Mayer-Salovey-Caruso Emotional Intelligence Test (MSCEIT) item booklet *(Version 2.0)*.

[B54-behavsci-15-00759] Ma Z., Tian Y., Li J., Liu J., Wang D., Zhang X. Y. (2024). Association of empathy with clinical symptoms and cognitive function in chronic schizophrenia patients with and without suicide attempts. European Archives of Psychiatry and Clinical Neuroscience.

[B57-behavsci-15-00759] McDonald S., Bornhofen C., Shum D., Long E., Saunders C., Neulinger K. (2006). Reliability and validity of The Awareness of Social Inference Test (TASIT): A clinical test of social perception. Disability and Rehabilitation.

[B58-behavsci-15-00759] Miller I. W., Norman W. H., Bishop S. B., Dow M. G. (1986). The modified scale for suicidal ideation: Reliability and validity. Journal of Consulting and Clinical Psychology.

[B59-behavsci-15-00759] National Institutes of Health (2014). Quality assessment tool for observational cohort and cross-sectional studies.

[B60-behavsci-15-00759] OCEBM Levels of Evidence Working Group (n.d.). The Oxford 2011 levels of evidence. Oxford centre for evidence-based medicine.

[B61-behavsci-15-00759] Page M. J., McKenzie J. E., Bossuyt P. M., Boutron I., Hoffmann T. C., Mulrow C. D., Shamseer L., Tetzlaff J. M., Akl E. A., Brennan S. E., Chou R. (2021). The PRISMA 2020 statement: An updated guideline for reporting systematic reviews. Bmj.

[B62-behavsci-15-00759] Parrish E. M., Pinkham A., Moore R. C., Harvey P. D., Granholm E., Roesch S., Joiner T., Depp C. A. (2024). An ecological momentary cognitive assessment study of over-attribution of threat and suicide risk factors in people with serious mental illness. Schizophrenia Research.

[B63-behavsci-15-00759] Pelizza L., Azzali S., Garlassi S., Scazza I., Paterlini F., Rocco Chiri L., Poletti M., Pupo S., Raballo A. (2020). Un estudio longitudinal de 2 años sobre la experiencia subjetiva de la cognición social en jóvenes con primer episodio de psicosis. Actas Españolas de Psiquiatria.

[B64-behavsci-15-00759] Peralta V., Cuesta M. J. (1994). Validación de la escala de los síndromes positivo y negativo (PANSS) en una muestra de esquizofrénicos españoles. Actas Luso Españolas de Neurología Psiquiatría y Ciencias Afines.

[B65-behavsci-15-00759] Pérez-Albéniz A., De Paúl J., Etxeberría J., Montes M. P., Torres E. (2003). Adaptación de interpersonal reactivity index (IRI) al español. Psicothema.

[B66-behavsci-15-00759] Phelan M., Slade M., Thornicroft G., Dunn G., Holloway F., Wykes T., Strathdee G., Loftus L., McCrone P., Hayward P. (1995). The Camberwell Assessment of Need: The validity and reliability of an instrument to assess the needs of people with severe mental illness. The British Journal of Psychiatry.

[B67-behavsci-15-00759] Pinkham A. E. (2014). Social cognition in schizophrenia. The Journal of Clinical Psychiatry.

[B68-behavsci-15-00759] Posner K., Brown G. K., Stanley B., Brent D. A., Yershova K. V., Oquendo M. A., Currier G. W., Melvin G. A., Greenhill L., Shen S., Mann J. J. (2011). The Columbia–Suicide Severity Rating Scale: Initial validity and internal consistency findings from three multisite studies with adolescents and adults. American Journal of Psychiatry.

[B69-behavsci-15-00759] Premack D., Woodruff G. (1978). Does the chimpanzee have a theory of mind?. Behavioral and Brain Sciences.

[B70-behavsci-15-00759] Roca M., Del Amo A. R.-L., Riera-Serra P., Pérez-Ara M. A., Castro A., Roman Juan J., García-Toro M., García-Pazo P., Gili M. (2019). Suicidal risk and executive functions in major depressive disorder: A study protocol. BMC Psychiatry.

[B71-behavsci-15-00759] Rocca P., Galderisi S., Rossi A., Bertolino A., Rucci P. A., Gibertoni D. I., Montemagni C., Sigaudo M., Mucci A., Bucci P., Acciavatti T. (2016). Social cognition in people with schizophrenia: A cluster-analytic approach. Psychological Medicine.

[B72-behavsci-15-00759] Rosales C., Torres F., Luna J., Jiménez J., Martínez G. (2002). Fiabilidad del instrumento de Evaluación de Necesidades de Camberwell (CAN)(Versión española del CAN Estudio de fiabilidad). Actas Españolas de Psiquiatría.

[B73-behavsci-15-00759] Sanz J., Perdigón A. L., Vázquez C. (2003). The Spanish adaptation of Beck’s Depression Inventory-II (BDI-II): 2. Psychometric properties in the general population. Clínica y Salud.

[B74-behavsci-15-00759] Sarró S., Dueñas R. M., Ramírez N., Arranz B., Martínez R., Sánchez J. M., González J. M., Saló L., Miralles L., San L. (2004). Cross-cultural adaptation and validation of the Spanish version of the Calgary Depression Scale for Schizophrenia. Schizophrenia Research.

[B75-behavsci-15-00759] Sastre-Buades A., Alacreu-Crespo A., Courtet P., Baca-Garcia E., Barrigon M. L. (2021). Decision-making in suicidal behavior: A systematic review and meta-analysis. Neuroscience and Biobehavioral Reviews.

[B76-behavsci-15-00759] Sastre-Buades A., Caro-Cañizares I., Ochoa S., Lorente-Rovira E., Barajas A., Gutiérrez-Zotes A., Sánchez-Alonso S., López-Carrilero R., Grasa E., Pousa E., Pélaez T., Cid J., González-Higueras F., Ruiz-Delgado I., Baca-Garcia E., Barrigon M. L. (2023). Relationship between cognition and suicidal behavior in recent-onset psychosis. Schizophrenia Research.

[B77-behavsci-15-00759] Savla G. N., Vella L., Armstrong C. C., Penn D. L., Twamley E. W. (2013). Deficits in domains of social cognition in schizophrenia: A meta-analysis of the empirical evidence. Schizophrenia Bulletin.

[B78-behavsci-15-00759] Turecki G., Brent D. A., Gunnell D., O’Connor R. C., Oquendo M. A., Pirkis J., Stanley B. H. (2019). Suicide and suicide risk. Nature Reviews Disease Primers.

[B79-behavsci-15-00759] Van’t Wout M., Aleman A., Kessels R. P., Larøi F., Kahn R. S. (2004). Emotional processing in a non-clinical psychosis-prone sample. Schizophrenia Research.

[B80-behavsci-15-00759] Vazquez-Barquero J. L., Gaite L., Arenal A., Díez Manrique J. F., Cuesta Núñez M., Higuera A. (1994). Development and verification of the Spanish version of the” scanning system” psychiatric interview (“Questionnaires for clinical evaluation in neuropsychiatry”. Actas Luso-Españolas de Neurología, Psiquiatría y Ciencias Afines.

[B81-behavsci-15-00759] Villa J., Ehret B. C., Depp C. A. (2019). Systematic review of the inclusion of people with psychosis in suicide-specific clinical trials. Crisis.

[B82-behavsci-15-00759] Villa J., Pinkham A. E., Kaufmann C. N., Granholm E., Harvey P. D., Depp C. A. (2018). Interpersonal beliefs related to suicide and facial emotion processing in psychotic disorders. Journal of Psychiatric Research.

[B83-behavsci-15-00759] Wang W., Zhou Y., Wang J., Xu H., Wei S., Wang D., Wang L., Zhang X. Y. (2020). Prevalence, clinical correlates of suicide attempt and its relationship with empathy in patients with schizophrenia. Progress in Neuro-Psychopharmacology and Biological Psychiatry.

[B84-behavsci-15-00759] Wastler H. M., Moe A. M., Pine J. G., Breitborde N. J. (2022). Cognition and suicide risk among individuals with first-episode psychosis: A 6-month follow-up. Psychiatric Rehabilitation Journal.

[B85-behavsci-15-00759] Westheide J., Quednow B. B., Kuhn K.-U., Hoppe C., Cooper-Mahkorn D., Hawellek B., Eichler P., Maier W., Wagner M. (2008). Executive performance of depressed suicide attempters: The role of suicidal ideation. European Archives of Psychiatry and Clinical Neuroscience.

[B86-behavsci-15-00759] Wing J. K., Babor T., Brugha T. S., Burke J., Cooper J. E., Giel R., Jablenski A., Regier D., Sartorius N. (1990). SCAN: Schedules for clinical assessment in neuropsychiatry. Archives of General Psychiatry.

[B87-behavsci-15-00759] World Health Organization (2014). Preventing suicide: A global imperative.

[B88-behavsci-15-00759] World Health Organization (2021). Suicide worldwide in 2019: Global health estimates.

[B89-behavsci-15-00759] Yin Y., Tong J., Huang J., Tian B., Chen S., Cui Y., An H., Tan S., Wang Z., Yang F., Tian L. (2020). Suicidal ideation, suicide attempts, and neurocognitive dysfunctions among patients with first-episode schizophrenia. Suicide and Life-Threatening Behavior.

[B90-behavsci-15-00759] Yüksel A., Yilmaz E. B., Dikmen S. N. (2024). Suicide probability among patients with schizophrenia and bipolar disorder. Journal of Psychosocial Nursing and Mental Health Services.

[B91-behavsci-15-00759] Zhu Q. Y., Zhang X. Y. (2024). Correlation analysis and gender differences of cognitive function based on mini-mental state examination (MMSE) and suicidal tendency in patients with schizophrenia. BMC Psychiatry.

