# Supplementary material for: Social Cognition in Suicidal Behavior in Psychosis: A Systematic Review
Source: Behav Sci (Basel). 2025 Jun 1;15(6):759. doi: 10.3390/bs15060759 (PMC12189955; doi:10.3390/bs15060759)
Supplement: Supplementary file 1 [file behavsci-15-00759-s001.zip › behavsci-3576079-supplementary.pdf]

Table S1. *Quality assessment of studies using a tool designed by the NIH (2014) for longitudinal and cross-sectional observational designs.*

	Research question	Study population	Study population	Eligibility criteria	Size justification	Exposure before measurement	Long term	Exposure levels	Measurements and evaluation	Repeated evaluation	Outcome measures	Assessors blinded	Follow-up	Statistical analysis
Abdo et al. (2021)	Yes	Yes	Yes	Yes	No	No	No	Yes	Yes	NA	Yes	NA	NA	No
Canal-Rivero et al. (2017)	Yes	Yes	Yes	Yes	Yes	Yes	Yes	Yes	Yes	No	Yes	NA	Yes	Yes
Chalker et al (2022)	Yes	Yes	Yes	No	Yes	No	No	Yes	Yes	NA	Yes	NA	NA	Yes
Comparelli et al. (2017)	Yes	Yes	Yes	NA	Yes	No	No	Yes	Yes	NA	Yes	NA	NA	Yes
Cuesta et al. (2022)	Yes	Yes	Yes	Yes	Yes	Yes	Yes	Yes	Yes	No	Yes	NA	No	Yes
Deep et al. (2018)	Yes	Yes	Yes	Yes	Yes	Yes	No	Yes	Yes	No	Yes	NA	Yes	Yes
Dickhoff et al. (2021)	Yes	Yes	Yes	NA	Yes	Yes	Yes	Yes	Yes	No	Yes	NA	Yes	Yes
Duñó et al. (2009)	Yes	Yes	Yes	Yes	Yes	No	No	Yes	Yes	NA	Yes	NA	NA	Yes
Harenski et al. (2017)	Yes	Yes	Yes	Yes	Yes	No	No	Yes	Yes	NA	Yes	NA	NA	Yes
Liu et al. (2023)	Yes	Yes	Yes	Yes	Yes	No	No	Yes	Yes	NA	Yes	NA	NA	Yes
Parrish et al. (2024)	Yes	Yes	Yes	No	Yes	Yes	No	Yes	Yes	Yes	Yes	NA	Yes	Yes
Rocca et al. (2016)	Yes	Yes	Yes	No	No	No	No	Yes	Yes	NA	Yes	NA	NA	No
Sastre-Buades et al. (2023)	Yes	Yes	Yes	Yes	Yes	No	No	Yes	Yes	NA	Yes	NA	NA	Yes
Villa et al. (2018)	Yes	Yes	No	Yes	Yes	No	No	Yes	Yes	NA	Yes	NA	NA	Yes
Wang et al. (2020)	Yes	Yes	Yes	Yes	Yes	No	No	Yes	Yes	NA	Yes	NA	NA	Yes
Wastler et al. (2022)	Yes	Yes	Yes	NA	Yes	Yes	Yes	Yes	Yes	Yes	Yes	NA	Yes	No
Yin et al. (2020)	Yes	Yes	No	Yes	Yes	No	No	Yes	Yes	NA	Yes	NA	NA	Yes
Zhu and Zhang (2024)	Yes	Yes	Yes	Yes	No	No	No	Yes	Yes	NA	Yes	NA	NA	Yes

Note: NA: Not applicable.
